# Adaptive Delay-Free Filtering Based on IMU for Improving Ship Heave Measurement

**DOI:** 10.3390/s23249791

**Published:** 2023-12-13

**Authors:** Daohua Lu, Yong Zhang, Jia Wang

**Affiliations:** 1 School of Mechanical Engineering, Jiangsu University of Science and Technology, Zhenjiang 212003, China; 211110201120@stu.just.edu.cn (Y.Z.); wjjzhb@just.edu.cn (J.W.); 2 Marine Equipment and Technology Institute, University of Science and Technology, Zhenjiang 212003, China

**Keywords:** IMU, adaptive digital filtering, heave motion, marine systems

## Abstract

Ship heave motion measurement is crucial for ensuring vessel stability, navigation precision, and maritime engineering safety. In order to achieve accurate heave motion measurement, a method based on an adaptive digital high–pass filter is proposed. The approach involves constructing a ship heave motion model, conducting an analysis of heave motion, determining the optimal cutoff frequency for the adaptive filter based on an analysis of filtering and sensor errors, and designing an adaptive delay–free digital high–pass filter. Through simulation experiments in various sea conditions and platform tests, the method demonstrates superior performance. In comparison to fixed–parameter complementary filters, it exhibits a reduction of over 50% in maximum error and mean square error.

## 1. Introduction

During ship navigation, various factors such as waves and wind induce six degrees [1] of freedom motion, with vertical heave motion being particularly critical. Measuring heave motion is essential for understanding the characteristics of ship stability, wave resistance, and maneuverability, contributing to the assessment of adaptability and safety under different sea conditions. This measurement not only aids in predicting potential issues but also provides reliable support for maritime trade and resource development, offering crucial information for decision−making and operations.

Measurements of heaving motion typically demand high precision, requiring accuracy at the centimeter level, as exemplified by methods such as barometric altimetry [2] and radio altimetry [3]. Currently, inertial navigation systems (INSs) are widely employed for heave motion measurement in maritime applications due to their outstanding autonomy and high precision. Grounded in Newton’s laws of motion, INSs integrate the output information from inertial measurement units (IMUs) to solve for heave motion parameters. However, during the computation of the vertical acceleration output from IMUs, challenges such as divergence and susceptibility to low−frequency signals may compromise measurement accuracy, leading to issues such as poor precision and phase advancement in the results [4].

Regarding the aforementioned issue, scholars mainly classify their research into two categories: one based on Kalman filtering and the other based on digital filters.

S. Küchler had used extended Kalman filtering to process acceleration signals and obtain heave information [5]. Guo proposed the use of a cubature Kalman filter (CKF) to address the high−dimensional nonlinear challenges in the ship heave motion model [6]. T. Zou et al. had proposed a self-adaptive robust volumetric Kalman filter based on the Sage–Husa noise estimator (SARCKF) for ship heave motion estimation [7]. But using Kalman filtering to process heave measurements has the disadvantage of high computational complexity, resulting in poor real-time performance and the need for external information assistance, which is difficult to meet at sea. Scholars have conducted extensive research and exploration on traditional digital filters. J.M. Godhavn proposed a fourth−order filter design algorithm suitable for heave motion measurements. By selecting appropriate filter cutoff frequencies, optimal measurement performance was achieved [8]. Yueyang Ben et al. proposed a bandpass filter, and a weighted frequency Fourier linear combiner (WFLC) method was designed as an adaptive frequency estimator. This method eliminates peak−to−peak errors and adds an addition filter to avoid phase correction [4]. X. Wei et al. had proposed an adaptive multiple low−pass filter to address the phase lead error [9].

The aforementioned authors made efforts to enhance heave measurement accuracy and reduce latency; however, they overlooked the performance and real−time aspects of heave measurements in dynamically changing sea conditions. To address this issue, this paper proposes a delay−free adaptive filtering approach, ensuring rapid and accurate measurement of heaving motion information under varying sea conditions. This paper first introduces the fundamental approach of utilizing inertial navigation for heave motion measurement in maritime vessels. Subsequently, a frequency domain analysis is conducted on heave acceleration to extract the frequency characteristics of heave motion, leading to the determination of the optimal cutoff frequency for filtering. Building upon this optimal cutoff frequency and employing a complementary approach, an adaptive filtering method is devised. Finally, experimental validation is conducted to assess the efficacy of the proposed methodology.

## 2. Principle of Heave Measurement

### 2.1. Heave Motion Model

**Figure 1:**
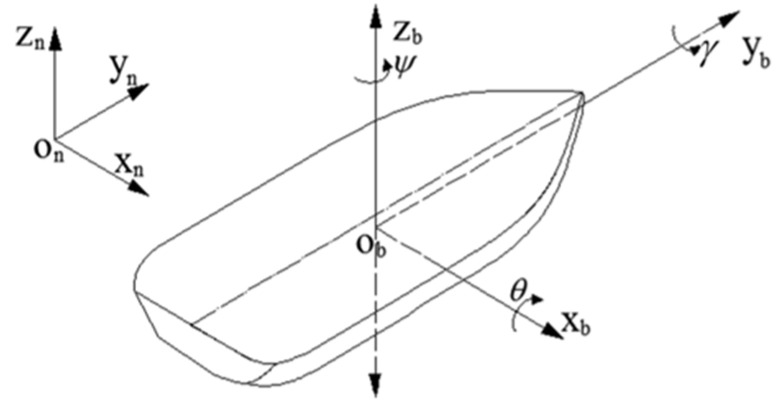
Schematic diagram of ship heave measurement.

Waves are a stochastic and complex fluctuation phenomenon, with their periods and amplitudes undergoing random variations influenced by changes in the sea wind. Under the influence of waves, vessels experience six−degrees−of−freedom motion, as illustrated in Figure 1. The heave motion of a vessel refers to the reciprocating movement along the longitudinal axis of the geographic coordinate system. This motion exhibits a relatively short period, typically ranging from a few seconds to several tens of seconds, and can thus be regarded as a high−frequency oscillation. According to the principle of signal superposition, the heave motion of a vessel can be approximated as the summation of multiple cosine waves with varying amplitudes and frequencies [10]:

(1) $$h(t)=\sum_{i=1}^{N}\left[A_{ei}\xi_{ai}\cos(\omega_{i}t+\varepsilon_{i}+\gamma_{ei})\right]\begin{array}{c} \underline{\underline{A_{i}=A_{ei}\xi_{ai}}}\\ \alpha_{i}=\varepsilon_{i}+\gamma_{ei} \end{array}\sum_{i=1}^{N}A_{i}\cos(\omega_{i}t+\alpha_{i})=\sum_{i=1}^{N}h_{i}(t)$$

where $A_{ei}$ and $\gamma_{ei}$ denote the amplitude and phase, respectively, of the transfer function for heave motion; $\xi_{ai}$, $\omega_{i}$ and $\varepsilon_{i}$ denote the amplitude, frequency, and initial phase, respectively, of the *i*−th cosine wave, respectively; *t* and *N* denote time, and the number of cosine waves.

### 2.2. Heave Motion Measurement Based on IMU

When measuring heave motion information of a vessel, the inertial measurement unit (IMU) is often installed at the center of the ship, with the ship’s body coordinate system defined as ‘right−front−up’ (*b*−frame or body coordinate system) and the geographic coordinate system defined as ‘east−north−up’ (*n*−frame or navigation coordinate system), as illustrated in Figure 1.

The inertial navigation system can measure the acceleration along the three axes of the ship’s coordinate system and the angular velocity around the three axes [11]. After information processing, the vertical acceleration of the ship can be obtained:

(2) v˙=Cbnfsfb−(2ω+ienωenn)×vn+gn

where $v^{n}$, $f_{sf}^{b}$, $g^{n}$, $C_{n}^{b}$, and $(2\omega_{ie}^{n}+\omega_{en}^{n})\times v^{n}$ denote the speed in the navigation coordinate system, acceleration in the carrier coordinate system, the projection of gravitational acceleration in the navigation coordinate system, attitude matrix, and the Earth’s rotation and Colombian compensation term, which can be ignored when the ship speed is small.

The above formula can be simplified as:

(3) $$\dot{v}=C_{b}^{n}f_{sf}^{b}+g^{n}$$

In summary, the acceleration in the heave direction of a ship can be expressed as:

(4) $$a_{h}=a_{z}-g^{n}-b-\varsigma$$

where $a_{z}$, b, and ς denote the measured values, constant bias, and the sensor’s inherent random noise error.

**Figure 2:**
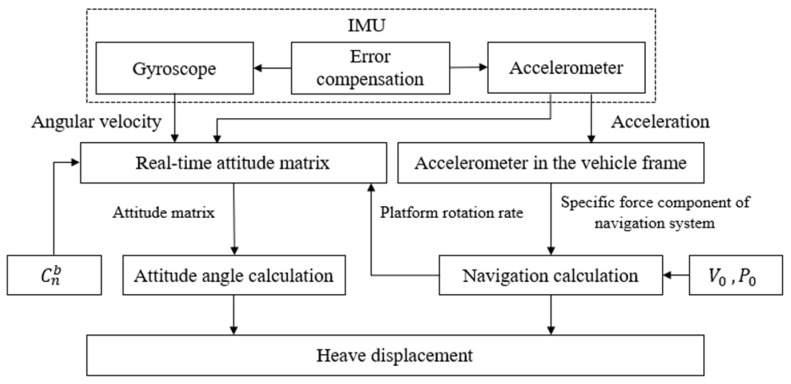
Inertial measurement unit (IMU) computation for heave displacement.

**Figure 3:**
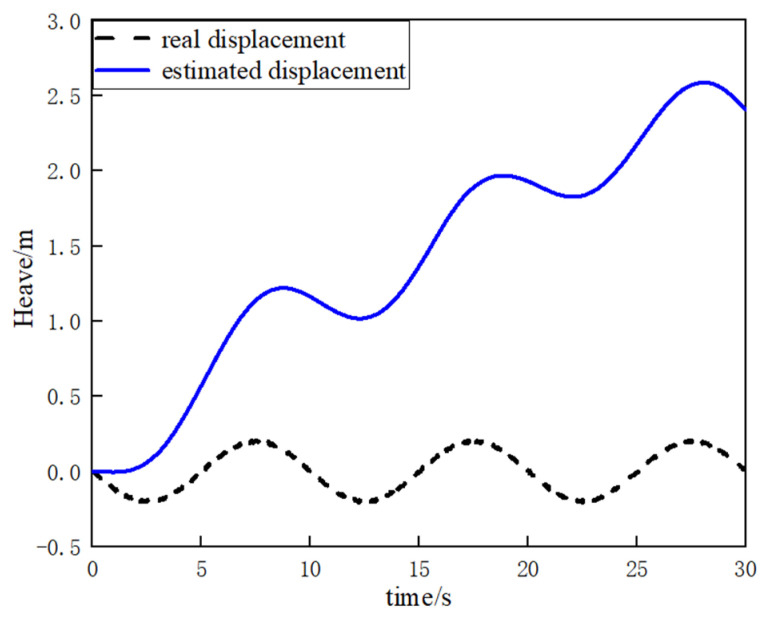
Comparison of a ship’s estimated and real displacement.

In theory, by integrating the erroneous heave acceleration obtained from IMU output twice, the heave displacement information of the vessel can be derived, as illustrated in Figure 2. However, the reality is often less than ideal, with significant discrepancies between estimated values and actual values, leading to persistent divergence and challenging convergence. The main cause of this discrepancy lies in directly integrating the output acceleration, neglecting the impact of errors and external disturbances. Over time, the calculated heave displacement deviates significantly from the true displacement, as depicted in Figure 3.

## 3. Filter Design

**Figure 4:**
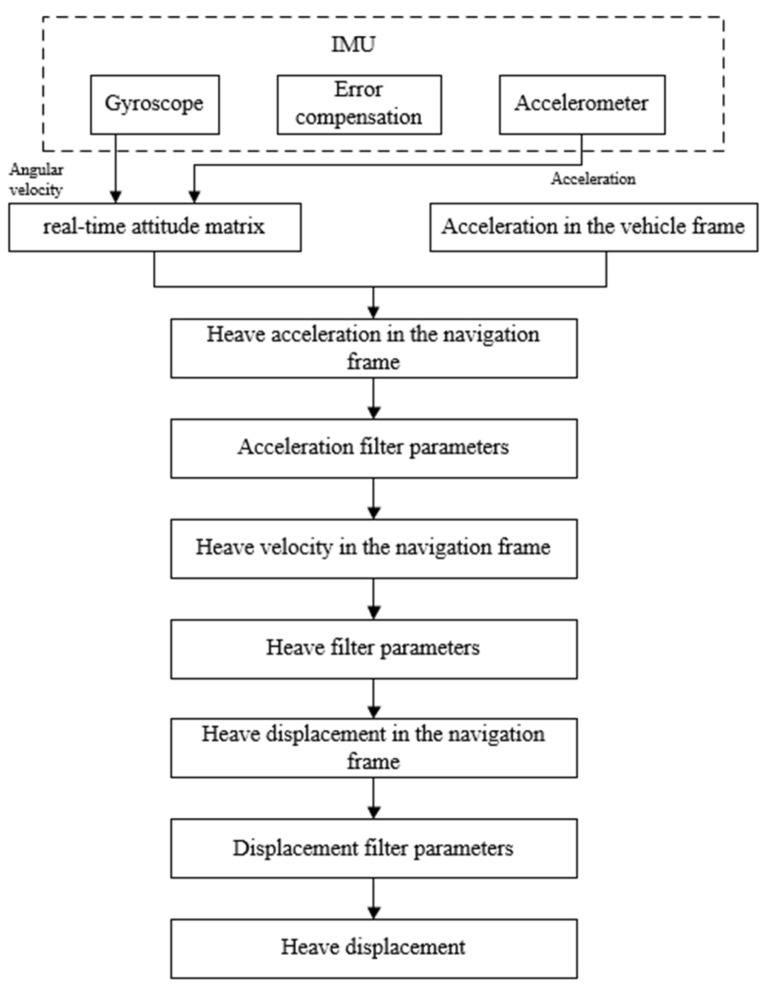
Flow chart of heave motion measurement algorithm.

As indicated earlier, directly integrating heave acceleration leads to a significant deviation between the output heave displacement and the actual values. Therefore, to obtain reliable and accurate heave displacement information, processing the vessel’s heave data is necessary. This paper employs a simple and reliable approach, using digital filters to eliminate detrimental signals in heave acceleration. The process involves obtaining heave acceleration data $a_{h}$ from the IMU output. Utilizing a fast Fourier transform (FFT), a filtering operation is applied to the acceleration data, followed by integration to derive heave velocity. Subsequently, the same steps are iterated to calculate heave displacement. The specific measurement process is illustrated in Figure 4.

### 3.1. Digital Filter

Processing the original output signal through a digital filter can effectively improve the accuracy of heave data. This article uses the Butterworth filter in IIR to process the original signal. System functions using second−order normalized Butterworth high−pass filters [12]:

(5) $$H(s)=\frac{s^{2}}{s^{2}+\sqrt{2}\omega_{c}s+\omega_{c}{}^{2}}$$

where s, and $\omega_{c}$ denote the complex frequency domain variable, and the cutoff frequency.

Furthermore, the ship heave error after filtering is expressed as:

(6) $$h_{1}(n)=h_{2}(n)-h(n)=(1-s^{2}H(s))h_{2}-H(s)(g+b+\zeta )$$

where $h_{2}(n)$ and h(n) denote the estimated value of ship heave displacement after filtering and the true value of ship heave displacement.

### 3.2. Error Analysis

As can be seen from the previous text, the cutoff frequency $\omega_{c}$ directly affects the measurement results in the heave error. To obtain the optimal filter parameters, consider the impact of errors on the filter.

#### 3.2.1. Filter Error

The heave motion of a ship can be formed by the superposition of multiple cosine waves with different amplitudes and frequencies, and the error of the filter is:

(7) $$e=\left\|1-sH(s)\right\|=\frac{\sqrt{\omega_{c}^{4}+2\omega_{i}^{2}\omega_{c}^{2}}}{\omega_{i}^{4}+\omega_{c}^{4}}\to \sqrt{2}\left(\frac{\omega_{c}}{\omega_{i}}\right),\omega_{i}>>\omega_{c}$$

The filter variance can be represented by the following equation:

(8) $$E[{\left(eh_{2}(n)\right)}^{2}]\leq 2A_{i}\left(\frac{\omega_{c}}{\omega_{i}}\right)$$

#### 3.2.2. Sensor Error

Similarly, the random error caused by sensor noise cannot be ignored. Assuming that the sensor noise is Gaussian white noise, its spectral density is:

(9) $$S(\omega )=\sigma^{2}$$

The sensor variance can be shown by the following equation [13]:

(10) $$\sigma_{i}^{2}=\frac{1}{2\pi }{\int_{-\infty }^{\infty }\left|H(\omega )\right|}^{2}S(\omega )d\omega =\frac{\sigma^{2}}{2^{3/2}\omega_{c}}$$

### 3.3. Adaptation of the Optimal Cutoff Frequency

In order to achieve optimal filtering performance, it is necessary to determine the optimal cutoff frequency to minimize the variance of the total error between the filter error and the sensor error [14]. The variance of the heave displacement error $h_{1}(n)$ can be expressed as

(11) $$\sigma_{h1(n)}^{2}=E[{\left(h_{1}(n)\right)}^{2}]=E[{\left(eh_{2}(n)\right)}^{2}]+\sigma_{i}{}^{2}\leq 2A_{i}\left(\frac{\omega_{c}}{\omega_{i}}\right)+\frac{\sigma^{2}}{2^{3/2}\omega_{c}}$$

From this, it can be concluded that when the error function is the minimum value, the following equation applies:

(12) $$\omega_{c}={\left(\frac{2^{3/2}\omega_{i}^{2}\sigma^{2}}{4A_{i}^{2}}\right)}^{1/3}$$

Under the action of waves, the frequency and amplitude of a ship will change, and the optimal cutoff frequency will also change with changes in $\omega_{i}$ and $A_{i}$. The fundamental frequencies $\omega_{i}$ and $A_{i}$ were determined using real−time fast Fourier transform and peak detection algorithms, thereby determining the optimal adaptive cutoff frequency under different sea conditions.

### 3.4. Design of Adaptive Filter

**Figure 5:**
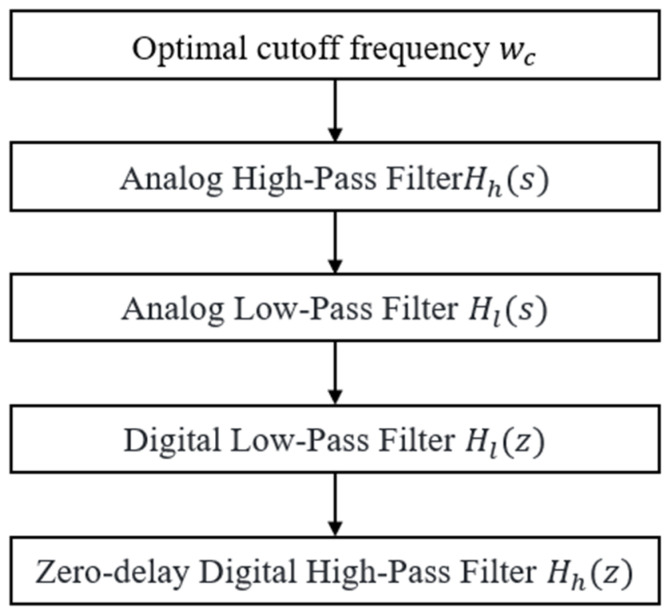
Flow chart of adaptive high pass filter algorithm.

Using the filter parameters obtained in the previous section, an analog low−pass filter a is obtained through the complementary idea. Through bilinear z−transform, the analog low−pass transfer function can be converted into the corresponding digital low−pass transfer function. Finally, using the complementary method again, the desired delay−free digital high−pass filter is obtained. The filter design is shown in Figure 5.

The amplitude and frequency of ship’s heave motion in the sea are not fixed. If a fixed−parameter high−pass filter is used, it will cause measurement errors. The algorithm proposed in this article obtains the frequency and wave height of ship’s heave motion through frequency analysis and obtains the real−time optimal cutoff frequency of the filter, so as to better calculate the ship’s heave information.

## 4. Heave Simulation Analysis

The following mainly studies the heave movement of ships under different sea conditions and designs experimental environments for ships in three different sea conditions.

**Figure 6:**
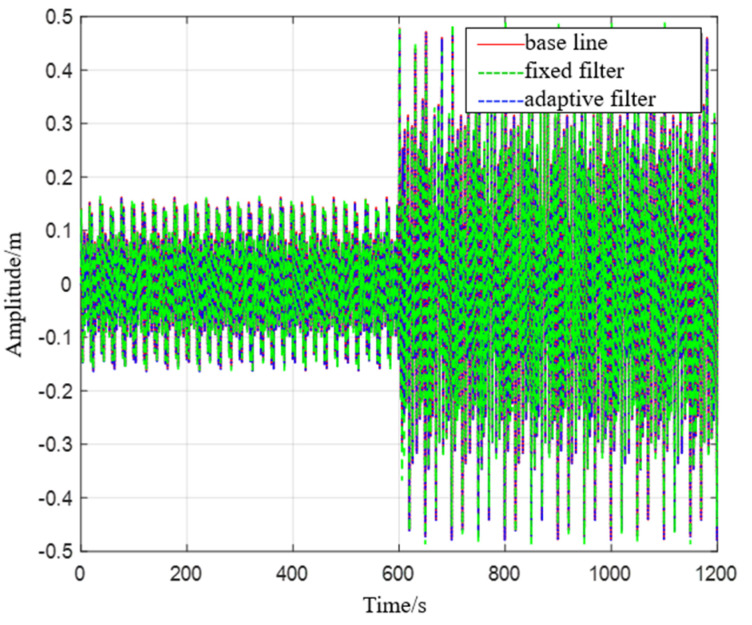
Comparison chart of level 1 to level 2 sea state rise and drop measurements.

Simulation 1: When the ship is in a moored state and moves in two different sea conditions, it moves for 10 min in the first sea condition and then moves for 10 min in the second sea condition (see Figure 6).

**Figure 7:**
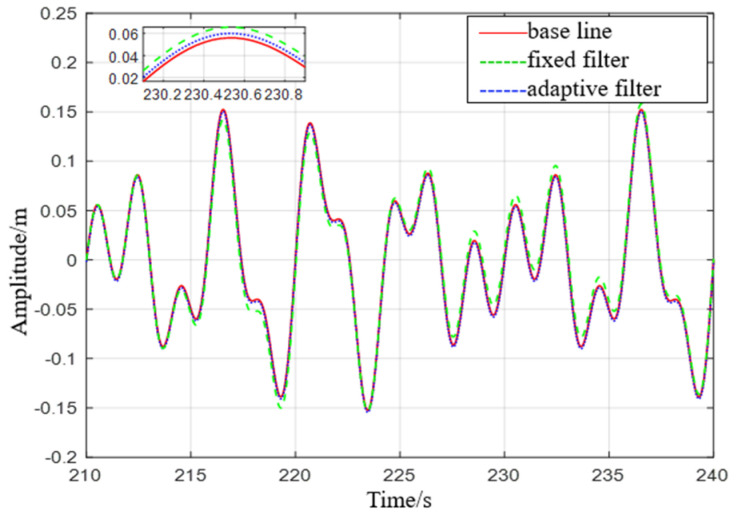
Comparison of level 1 sea conditions.

**Figure 8:**
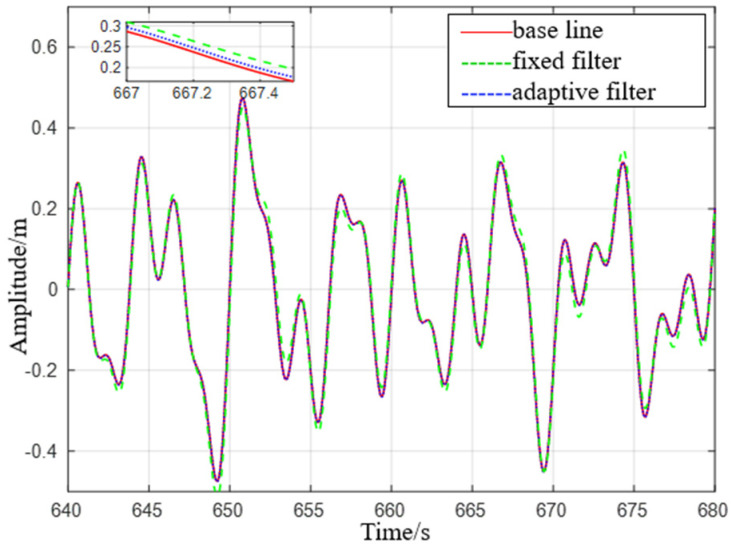
Comparison of level 2 sea conditions.

It can be seen from Figure 7 that the adaptive delay−free digital high−pass filter matches the reference value, and the fixed−parameter complementary high−pass filter matches the reference value overall, but there are some errors at the peak. After the sea condition changes, the adaptive delay−free digital high−pass filter matches the reference value in Figure 8, but the fixed−parameter complementary high−pass filter has a large error in the time period of 675 s to 680 s, and the performance is poor.

**Figure 9:**
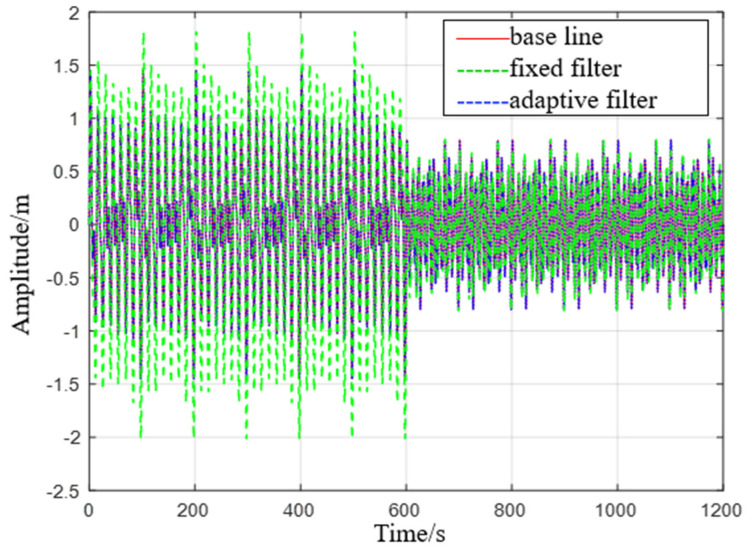
Comparison chart of level 5 to level 4 sea state rise and drop measurements.

Simulation 2: When the ship is in a moored state and moves in two different sea conditions, it moves for 10 min in the fifth sea condition and then moves for 10 min in the fourth sea condition (see Figure 9).

**Figure 10:**
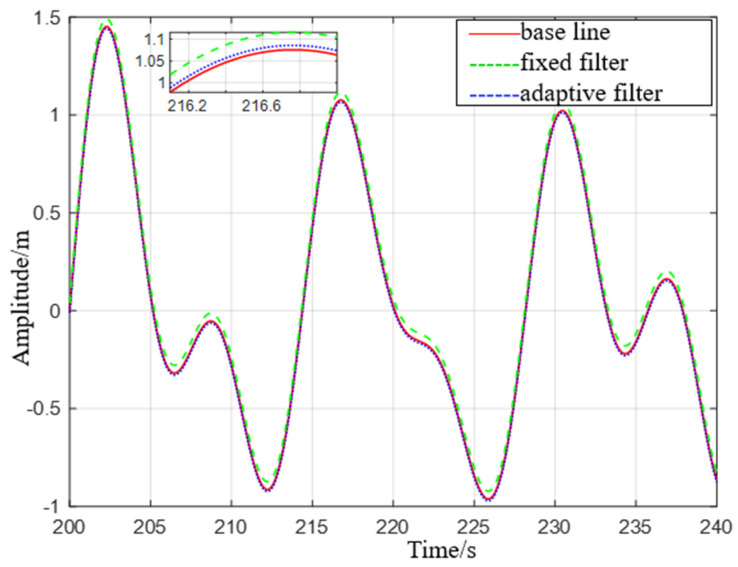
Comparison of level 5 sea conditions.

**Figure 11:**
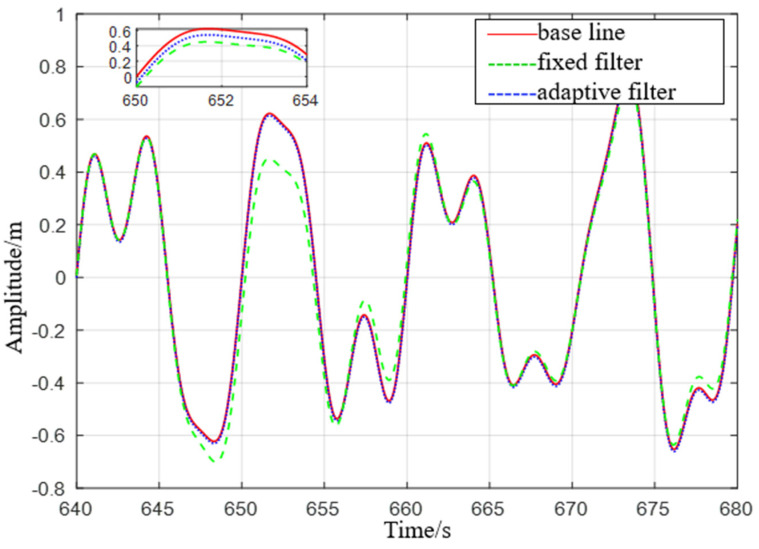
Comparison of level 4 sea conditions.

It can be seen from Figure 10 that the adaptive delay−free high−pass filter matches the reference value, and the fixed−parameter complementary high−pass filter matches the reference value overall, but there are some errors at the peak. After the sea condition changes, it can be seen from Figure 11 that the adaptive delay−free high−pass filter also matches the reference value, but the fixed−parameter complementary high−pass filter has a large error in the time period of 645 s to 655 s, and the performance is poor.

**Figure 12:**
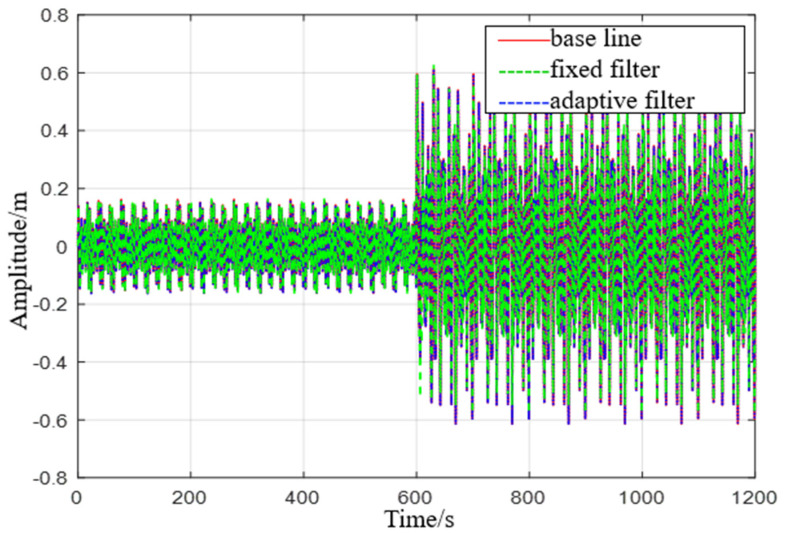
Comparison chart of level 1 to level 3 sea state rise and drop measurements.

Simulation 3: When the ship is in a uniform motion state and moves in two different sea conditions, it moves for 10 min in the first level sea state and then moves for 10 min in the third level sea state (see Figure 12).

**Figure 13:**
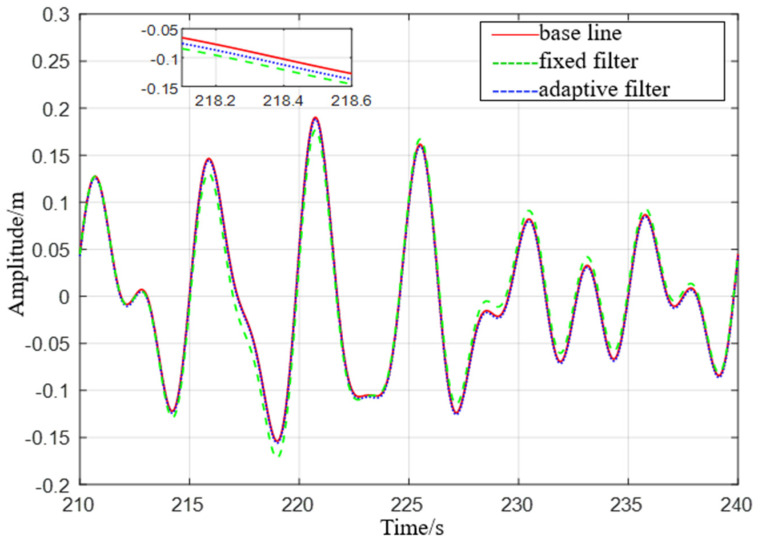
Comparison of level 1 sea conditions.

**Figure 14:**
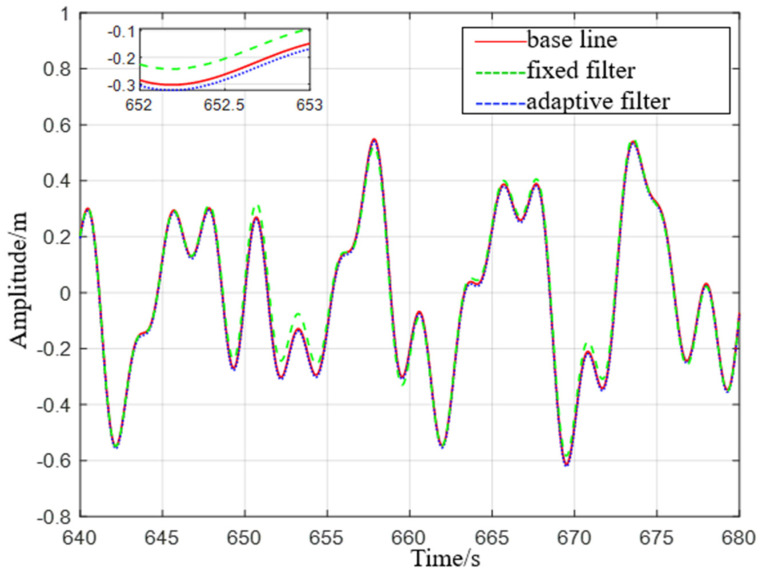
Comparison of level 3 sea conditions.

It can be seen from Figure 13 that the adaptive delay−free digital high−pass filter matches the reference value, and the fixed−parameter complementary high−pass filter matches the reference value overall, but there are some errors at the peak. After the sea condition changes, it can be seen from Figure 14 that the adaptive delay−free digital high−pass filter also matches the reference value, but the fixed−parameter complementary high−pass filter has a large error in the time period of 650 s to 655 s, and the performance is poor.

Based on the simulation experimental results, it can be observed that compared with the reference value, the adaptive delay−free digital high-pass filter obtained a more accurate estimation of the ship’s heave displacement. Although the fixed−parameter complementary high−pass filter can better approximate the true displacement, there are errors at the peak, and after the sea condition changes, there are large errors in some frequency bands. Therefore, the adaptive delay−free digital high−pass filter can adaptively adjust with the amplitude and frequency of the heave movement, resulting in higher estimation accuracy and a stronger ability to adapt to changes in wave amplitude and period.

## 5. Platform Experimental Verification

**Figure 15:**
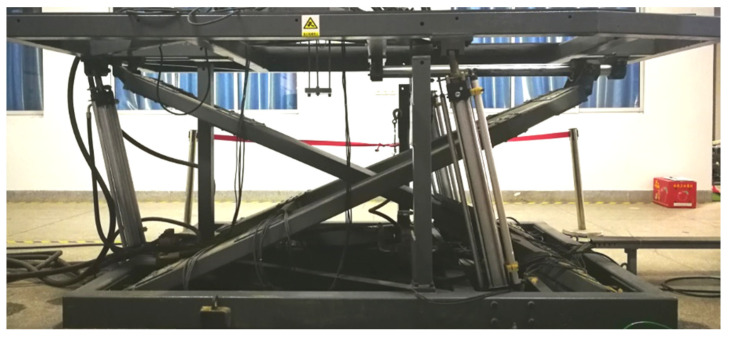
Sea state simulation platform.

**Figure 16:**
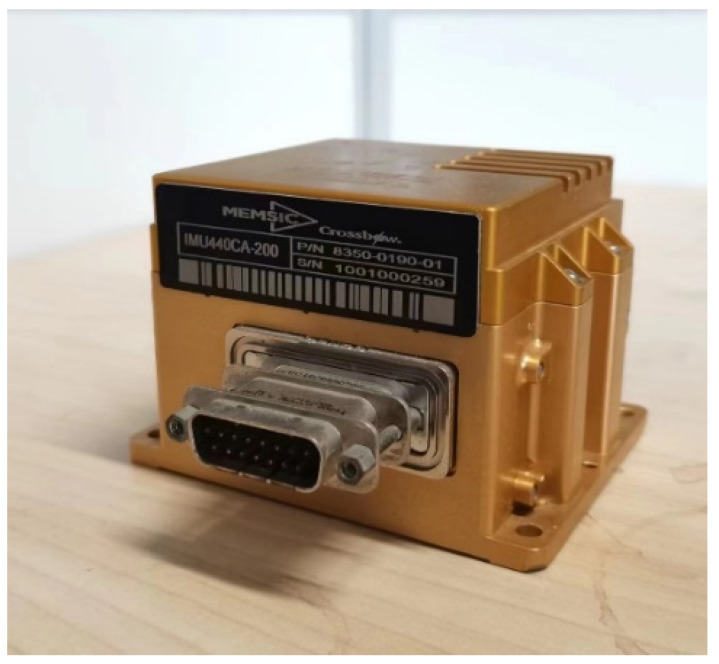
IMU440.

To verify the algorithm proposed in this article, platform experiments were conducted. The main equipment used was a three−degrees−of−freedom motion platform that simulated different sea conditions, an IMU440 (MEMSIC, Andover, MA, USA) that measured heave information, and a laser ranging instrument that provided the benchmark measurement, as shown in Figure 15 and Figure 16. Table 1 and Table 2 list the parameters of the equipment. The IMU440 is mounted on the installation platform of a three−degrees−of−freedom motion platform, situated at the central position of the platform. The laser rangefinder is oriented downward, providing distance information relative to the fixed ground platform. To minimize the impact of external factors, both measurement devices are installed in parallel.

We used Experiment 1 as the reference sea state and Experiment 2 and Experiment 3 as the comparison group. In Experiment 2 and Experiment 3, the filter parameters used were consistent.

**Figure 17:**
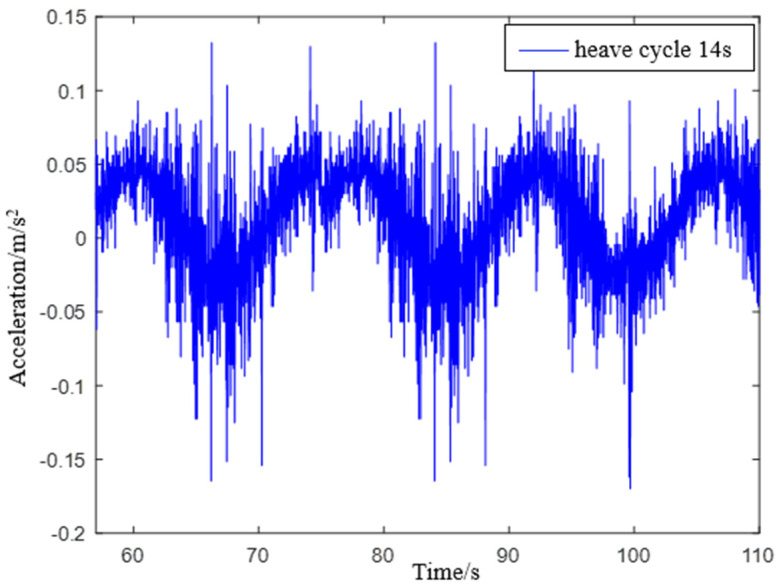
IMU440 measurement data with a period of 14 s.

**Figure 18:**
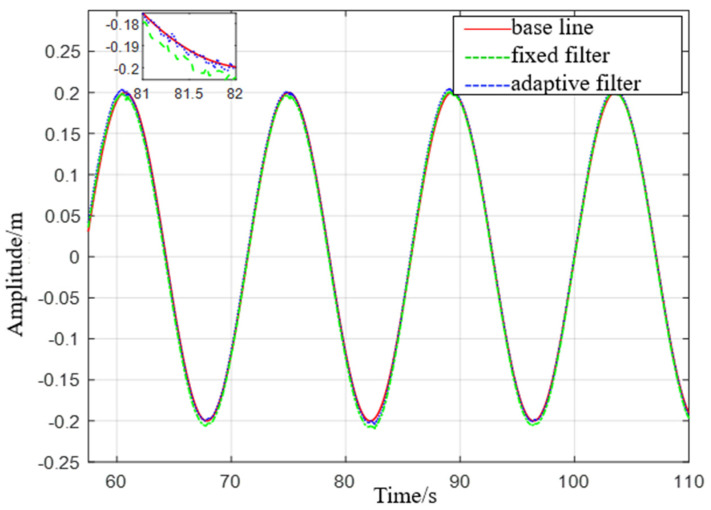
Comparison diagram of displacement with a heave period of 14 s.

**Experiment 1.**  *Simulate a wave heave motion with a maximum amplitude of approximately 0.20 m and a period of approximately 14 s for 600 s. Collect experimental data using IMU440, select the acceleration in the heave direction during the 54–110 s period as shown in Figure 17, and compare the filtering results as shown in Figure 18.*

**Figure 19:**
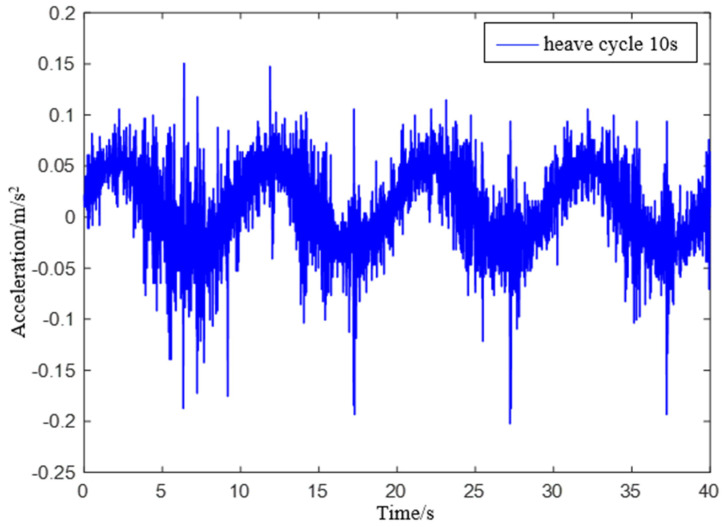
IMU440 measurement data with a period of 10 s.

**Figure 20:**
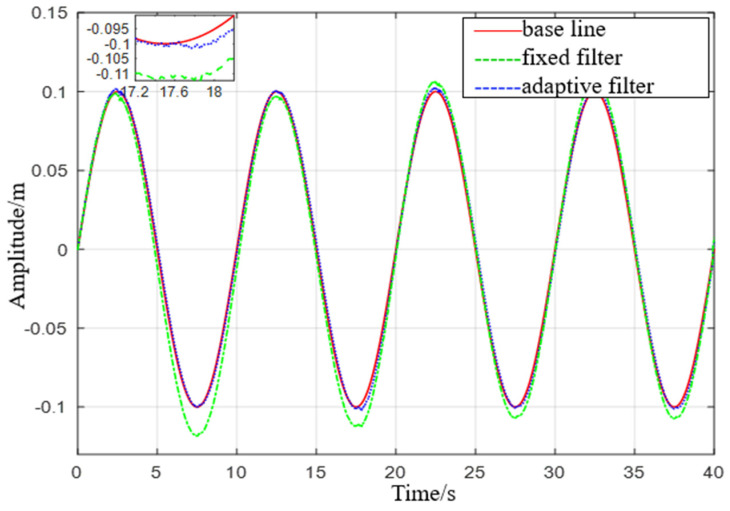
Comparison diagram of displacement with a heave period of 10 s.

Experiment 2: Simulate a wave heave motion with a maximum amplitude of 0.1 m and a period of 10 s for 600 s. Collect experimental data using IMU440, select the acceleration in the heave direction during the 0−40 s period as shown in Figure 19, and compare the filtering results as shown in Figure 20.

**Figure 21:**
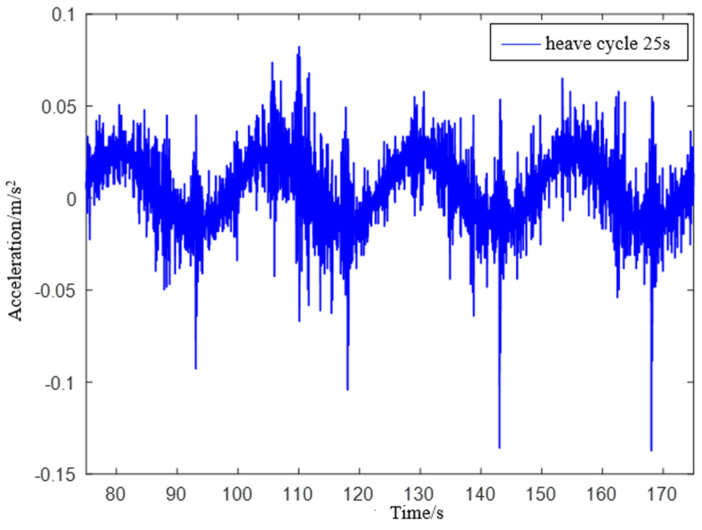
IMU440 measurement data with a period of 25 s.

**Figure 22:**
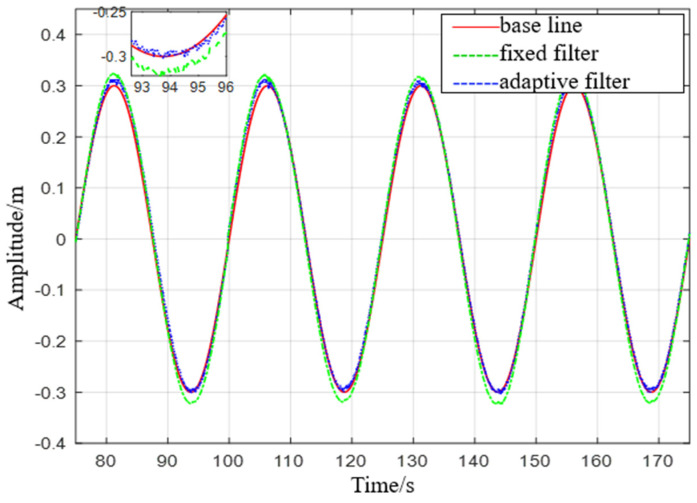
Comparison diagram of displacement with a heave period of 25 s.

Experiment 3: Simulate a wave heave motion with a maximum amplitude of 0.3 m and a period of 25 s for 600 s. Collect experimental data using IMU440, select the acceleration in the heave direction during the period of 75−175 s as shown in Figure 21, and compare the filtering results as shown in Figure 22.

In order to provide a more intuitive analysis of the experimental results, a precision statistical comparison was conducted between the fixed−parameter complementary high pass filter and the filter proposed in this paper, as shown in Table 3.

There is a table above that can be analyzed to determine:

(1) From Experiment 1, it can be seen that the maximum error of the fixed−parameter complementary high−pass filter is 0.0119 m, and the mean square error is 0.0096 m. However, the maximum error estimated by the adaptive filter is 0.0097 m, and the mean square error is 0.0096 m. Compared to fixed-parameter complementary high−pass filters, the adaptive filter demonstrates a reduction in both maximum error and mean square error in estimation. This indicates its superior performance in enhancing measurement accuracy and reducing estimation errors.

(2) From Experiment 2, it is evident that the maximum error of the fixed−parameter complementary filter treatment, 0.0269 m, is approximately six times higher than that of the proposed filter treatment in this study. The mean square error of the fixed−parameter complementary filter treatment, 0.0077 m, is about twice as high as that of the proposed filter treatment. When sea conditions change, this filter demonstrates higher accuracy and applicability compared to the proposed filter in this study.

(3) From Experiment 3, it can be seen that the maximum error of the fixed−parameter complementary filter treatment is about twice as high as that of the proposed filter treatment, at 0.0231 m. The mean square error of the fixed−parameter complementary filter treatment is about 1.2 times higher than that of the proposed filter treatment, at 0.0113 m.

(4) In conclusion, from the aforementioned experiments, it is evident that under similar sea conditions, the proposed filter in this study performs slightly better than the fixed−parameter complementary filter, although the difference is not significant. However, when sea conditions change, the fixed−parameter complementary filter exhibits limited adaptability, while the proposed filter proves more effective. The proposed filter demonstrates adaptive tuning with the amplitude and frequency of heave motion, resulting in higher estimation accuracy and a stronger ability to adapt to changes in wave amplitude and period.

## 6. Conclusions

In a maritime environment, external factors such as waves can induce six-degrees-of-freedom motion in ships during operations. Among these movements, vertical heave motion plays a pivotal role in material supply between ships, making accurate measurement crucial. To address issues encountered in traditional filters, such as phase lead and amplitude errors, this paper proposes a design approach for an adaptive digital filter. This method involves conducting frequency domain analysis on heave acceleration to derive the frequency characteristics of heave motion. Subsequently, it determines the optimal cutoff frequency for the filter. Building upon the optimal cutoff frequency and complementary principles, an adaptive digital high-pass filter is then designed.

The results indicate that the proposed adaptive digital filter design method in this study effectively enhances measurement accuracy. Additionally, when sea conditions change, the method dynamically adjusts to accommodate variations in wave amplitude and frequency, thereby providing more accurate estimation results. This approach holds significant engineering application value and can serve as a valuable reference for the maritime operations of vessels at sea.

## Figures and Tables

**Table 1:** Platform parameters.

Technical Parameter	Range	Technical Parameter	Range
Lifting height	±375 mm	Pitch angular acceleration	$300{}^{\circ}/s^{2}$
Vertical direction speed	450 mm/s	Roll angle	±27°
Vertical acceleration	≥0.5 g	Roll angular velocity	±25°/s
Pitch angle	±22°	Roll angle acceleration	$300{}^{\circ}/s^{2}$
Pitch angular velocity	±22°/s	Platform size	3510(L) × 2440(W) × 1380(H)mm

**Table 2:** IMU440 parameters.

Technical Indicators	Gyroscope	Accelerometer
Dynamic measurement range	±400°/s	±10 g
Zero bias stability	±22°/h	1 mg
Zero deviation error (full temperature)	±0.2°/s	4 mg
Random walk	4.5°/√h	1 m/s/√h
Scale factor nonlinearity	0.5%fs	1%fs

**Table 3:** Platform experiment heave accuracy statistics.

Experiment Number	Fixed Filter		Adaptive Filter	
	Maximum Error (m)	Mean Square Error (m)	Maximum Error (m)	Mean Square Error (m)
1	0.0119	0.0096	0.0097	0.0075
2	0.0269	0.0077	0.0041	0.0037
3	0.0231	0.0113	0.0126	0.0095

## Data Availability

Not applicable.
